# Ultrasonic renal length as an indicator of renal fibrosis severity in non-diabetic patients with chronic kidney disease

**DOI:** 10.1007/s10157-024-02598-0

**Published:** 2024-11-19

**Authors:** Ziman Chen, Jun Jiang, Simon Takadiyi Gunda, Xinyang Han, Chaoqun Wu, Michael Tin Cheung Ying, Fei Chen

**Affiliations:** 1 Department of Health Technology and Informatics, The Hong Kong Polytechnic University, 11 Yuk Choi Rd, Hung Hom, 999077 Kowloon, Hong Kong; 2 Department of Radiology, The First Affiliated Hospital of Shenzhen University, Shenzhen Second People’s Hospital, Shenzhen, China; 3 Department of Ultrasound, The Fifth Affiliated Hospital of Sun Yat-Sen University, 52 Meihua East Rd, Zhuhai, 519000 China

**Keywords:** Chronic kidney disease, Fibrosis, Ultrasound, Renal length

## Abstract

**Background:**

Debate continues regarding the potential of the ultrasonic renal length to serve as an indicator for evaluating the advancement of renal fibrosis in chronic kidney disease (CKD). This study investigates the independent association between renal length and renal fibrosis in non-diabetic CKD patients and assesses its diagnostic performance.

**Methods:**

From April 2019 to December 2021, 144 non-diabetic patients diagnosed with CKD who underwent a renal ultrasound examination and kidney biopsy were prospectively enrolled. Patients were categorized into the mild fibrosis group (*n* = 70) and the moderate-severe group (*n* = 74) based on the extent of fibrotic involvement. Ultrasonic renal length was measured from pole-to-pole in the coronal plane. A receiver operating characteristic (ROC) curve, multivariable logistic regression analysis, and a generalized additive model were performed.

**Results:**

A negative linear correlation was found between renal length and moderate-severe renal fibrosis risk. Each centimeter increase in renal length decreased the odds of moderate-severe fibrosis by 38% (OR: 0.62; 95% CI 0.41–0.93; *P* = 0.020). After adjusting for confounders, the relationship persisted (OR: 0.58; 95% CI 0.33–1.00; *P* = 0.048). However, renal length presented limited discrimination ability in distinguishing degrees of renal fibrosis while controlling the key confounding factors, yielding an area under the ROC curve of only 0.58 (95% CI 0.45–0.70).

**Conclusion:**

While an inverse relationship exists between renal length and risk of having moderate-severe renal fibrosis in non-diabetic CKD patients, renal length alone is insufficient for diagnosing fibrosis severity, underscoring the need for additional diagnostic parameters in CKD assessment.

**Supplementary Information:**

The online version contains supplementary material available at 10.1007/s10157-024-02598-0.

## Introduction

Chronic kidney disease (CKD) is defined as a group of kidney disorders characterized by abnormalities in kidney structure or a decline in kidney function [1, 2]. When it deteriorates to end-stage renal disease (ESRD), dialysis or kidney transplantation are currently the only definitive therapeutic approach [3]. Renal fibrosis, regardless of potential etiologies, is the final pathologic pathway common in CKD exacerbation to ESRD [4–6]. Indeed, it has been identified as one of the most significant risk factors for CKD progression [7]. Thus, the precise assessment of renal fibrosis is of clinical significance in medical management or even in evaluating therapeutic responses.

Currently, histopathological examination of renal biopsies remains the gold standard for renal fibrosis diagnosis and staging [8, 9]. However, due to the inherent invasiveness of the procedure, a renal biopsy cannot be used to dynamically monitor disease progression in the course of treatment or prognostic decisions. Not only that, it carries up to a 10% risk of major complications, including infection, bleeding, and the development of an arteriovenous fistula [10, 11]. Compared with histopathological examination, conventional renal ultrasound (US) represents the first-line diagnostic tool for the assessment of CKD as it possesses unique features including noninvasiveness, ionization-free radiation, cost-effectiveness, and high portability [12, 13]. US parameters such as renal size and echogenicity differences within the parenchyma are used to identify CKD severity [14, 15]. Indeed, renal length is universally reported and is usually the only measurement given at renal US [16]. However, the diagnostic value of renal length demonstrated by previous studies is essentially established at the CKD stage, derived from estimated glomerular filtration rate (eGFR) but not pathologic evidence, and even the results of some studies contradict each other, which may be attributed to potential confounders not controlled for [14, 17, 18]. Thus, in this study, we aim to investigate the true association between ultrasonic renal length and pathology-based renal fibrosis in CKD, after adjusting for potential clinical and laboratory features.

## Materials and methods

### Ethics and patient enrollment

The protocol for this study was approval by the Ethics Committee of the institution and adhered to the Helsinki Declaration. Written informed consent was obtained from each subject prior to participation.

The prospective study was conducted between April 2019 and December 2021 among patients with CKD who underwent renal biopsies and renal US examinations. Following were the inclusion criteria for patient enrollment: (1) patients diagnosed with CKD based on the presence of kidney damage (determined by abnormalities in blood or urine composition, kidney biopsy findings, or radiographic abnormalities) or an eGFR lower than 60 ml/min/1.73 m^2^, for a duration of over 3 months, as specified in the Kidney Disease Improving Global Outcomes (KDIGO) 2012 guideline [19]; (2) patients undergoing renal US examination before renal biopsy; (3) patients with complete clinical information and laboratory data (sex, age, body mass index (BMI); comorbidities including hypertension and cardiovascular disease; blood urea nitrogen (BUN) and urine protein to creatinine ratio (UPCR)); and (4) patients with renal fibrosis staging scheduled for renal biopsy assessment. Exclusion criteria were as follows: (1) patients with an inadequate biopsy sample (a length of less than 10 mm or a number of glomeruli of less than 10); (2) patients with multiple renal cysts, masses, hydronephrosis, or calculi that could affect measurements of ultrasonic parameters; (3) patients with diabetes mellitus or amyloidosis, as kidney size may not decrease despite the presence of CKD and associated fibrosis. The indications for renal biopsy include persistent proteinuria, hematuria, and/or renal insufficiency, particularly when a pathologic diagnosis or further clarification of the underlying pathology is required.

### Renal US examination

**Fig. 1:**
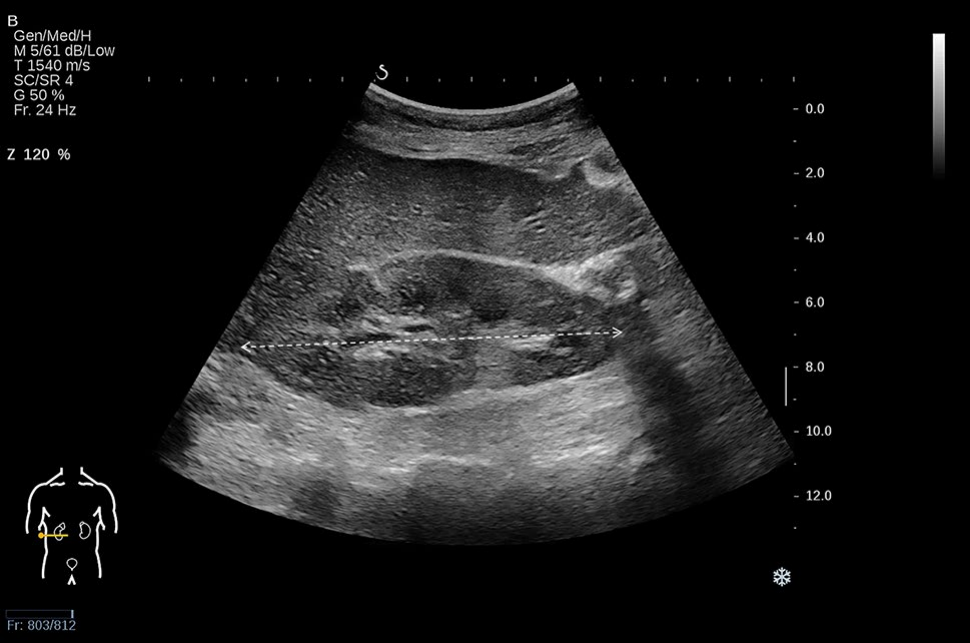
Longitudinal sonogram of the right kidney in a non-diabetic patient with chronic kidney disease. The dotted line indicates the maximal kidney length

Patients underwent a renal US examination two days prior to the renal biopsy. Using the Aixplorer US imaging system (SuperSonic Imagine, Aix-en-Provence, France) with a convex array probe (SC6-1, 1–6 MHz), a board-certified radiologist with 6 years’ experience interpreting abdominal US data independently performed all renal US examinations. The radiologist was unaware of the patients’ clinical characteristics. Participants assumed a supine position and breathed naturally. Conventional B-mode US was applied to scan the right kidney, and then renal length was measured from the upper pole to the lower pole at the coronal orientation; these measurements were repeated three times with an arithmetical mean for the final analysis (Fig. 1). Considering right kidney biopsy was performed on the patients, we chose to analyze measurements from the right kidney to ensure good alignment with the pathologic grading of renal fibrosis.

### Renal biopsy

Using a 16- or 18-gage needle (Bard Magnum, Covington, GA, USA), a percutaneous renal biopsy was performed at the lower pole of the right kidney. Pathologic specimens were evaluated independently by two pathologists with 6–8 years’ experience in nephropathology who were blinded to clinical information. Consensus was reached by resolving discrepancies through discussion. Renal fibrosis evaluation utilized a semi-quantitative scoring system, which considered pathologic observations encompassing glomerular characteristics (hypercellularity, segmental lesions, and global sclerosis), tubulointerstitial features (infiltration, fibrosis, and atrophy), and vascular changes (wall thickening and hyaline alterations). This scoring system was previously delineated in our study [20]. Subsequently, fibrosis severity was determined by stratifying cases into three distinct categories: mild, moderate, and severe.

### Statistic analysis

Statistic analysis was conducted using R software (version 4.2.1) and SPSS software (version 26.0; SPSS, Inc., Chicago, IL). Categorical variables were presented as counts (percentages), and continuous variables were expressed as mean ± standard deviation or median (interquartile range), as appropriate. The comparison of categorical variables was conducted using the Chi-squared or Fisher’s exact test, whereas the comparison of continuous variables employed either the Student’s *t* test or the Kruskal–Wallis test. In the present study, three models were proposed: the crude model (model 1), the minimally adjusted model (model 2), and the fully adjusted model (model 3). Model 1 did not involve any covariate adjustments. Model 2 solely accounted for demographic characteristics such as sex, age, and BMI. Model 3 was further adjusted to account for more covariates. These included laboratory indicators (BUN and UPCR), demographic factors (sex, age, and BMI), and comorbidities including hypertension and cardiovascular disease. The exploration of the correlation between renal length and the risk of moderate-severe renal fibrosis was conducted using multivariable logistic regression and the generalized additive model (GAM) methodologies [21–26]. To enhance the characterization of the relationship between the variables, a smooth curve fitting procedure, employing the penalized spline method within the GAM, was employed across three models. To analyze nonlinearity, an inflection point was initially estimated using a recursive algorithm, which was guided by the principle of maximizing the model’s goodness-of-fit. Starting with the 5th percentile of renal length data, candidate points were evaluated at 5% intervals, with corresponding likelihood values calculated for each. The renal length that yielded the best fit was identified as a potential inflection point. This search was then refined by exploring within a ± 5% range around the initial candidate point to further optimize the goodness-of-fit. Through iterative refinement, the exact location of the optimal inflection point was determined, potentially indicating critical changes in renal length related to the risk of moderate-severe renal fibrosis. The identification of this inflection point suggests that the rate of fibrosis risk may vary across different ranges of renal length. Subsequently, a two-piecewise linear regression model was constructed on both sides of the determined inflection point. The log-likelihood ratio test is employed as a statistical tool for assessing the goodness-of-fit between two models: the linear regression model and the two-piecewise linear regression model. In this step, the more complicated two-piecewise linear regression model, which encompasses additional parameters, is compared to the simpler linear regression model to see whether the more complicated model fits a specific dataset better. If the test result is not significant, it indicates that the additional parameters within the more intricate model are unsuitable for analyzing the current dataset and should be excluded from subsequent analyses. The log-likelihood ratio test serves a crucial role in assessing goodness-of-fit and aiding in the selection of the most suitable model for further analysis. To mitigate the impact of confounding factors, a covariate-adjusted receiver operating characteristic (ROC) curve was constructed for each of the three models to assess the diagnostic utility of renal length in distinguishing the severity of renal fibrosis. The covariate-adjusted ROC curve is computed as a weighted average of individual ROC curves corresponding to distinct covariate values, where the weights are determined by the proportion of target event occurrences stratified within each covariate value [27, 28]. Discriminative ability was quantified by the area under the ROC curve (AUC). The 95% confidence interval (CI) for the AUC value was derived employing the 500-bootstrap resampling technique [29]. Through the generation of 500 bootstrap samples obtained by randomly selecting observations from the original population with replacement, a diverse array of resampled datasets was obtained. For each bootstrap sample, the relevant statistic of interest was computed. Subsequently, through repeated resampling and estimation iterations, the resulting estimated statistic values were amalgamated into a comprehensive dataset, facilitating the calculation of the sampling distribution. Sensitivity, specificity, and accuracy were determined by the optimal cut-off value using the Youden index [30]. For all the analyses, a two-sided *P* value less than 0.05 denoted statistical significance.

## Results

### Patient characteristics

A total of 144 patients who met the eligibility criteria were included in this study. Based on their histologic scores, the patients were stratified into three categories: mild fibrosis (*n* = 70), moderate fibrosis (*n* = 62), and severe fibrosis (*n* = 12). Due to the relatively low number of severe cases, the moderate and severe groups were combined into a single group for subsequent analysis, referred to as the “moderate-severe” group in comparison to the “mild” group. The average age of the moderate-severe fibrosis group (44.51 ± 13.81 years) was significantly higher than that of the mild fibrosis group (33.94 ± 12.70 years) (*P* < 0.001). The mild fibrosis group exhibited a higher eGFR (107.89 (90.48–120.61) ml/min/1.73 m^2^) compared to the moderate-severe fibrosis group (67.30 (48.11–100.48) ml/min/1.73 m^2^) (*P* < 0.001). Furthermore, the mild fibrosis group had significantly greater ultrasonic renal length (10.61 ± 0.77 cm) than the moderate-severe fibrosis group (10.28 ± 0.90 cm) (*P* = 0.018). Detailed baseline characteristics of the enrolled participants are presented in Table 1, while the etiology of CKD is outlined in Table S1.

**Table 1:** Baseline characteristics of participants (*N* = 144)

Characteristic	Mild fibrosis (*N* = 70)	Moderate-severe fibrosis (*N* = 74)	*P* value
Age (years)	33.94 ± 12.70	44.51 ± 13.81	< 0.001
Sex			
Male	41 (58.57%)	37 (50.00%)	0.302
Female	29 (41.43%)	37 (50.00%)	
BMI (kg/m^2^)	24.39 ± 4.18	23.60 ± 3.37	0.211
eGFR (ml/min/1.73m^2^)	107.89 (90.48–120.61)	67.30 (48.11–100.48)	< 0.001
Blood urea nitrogen (mmol/L)	4.52 (3.60–5.59)	5.75 (4.83–7.59)	< 0.001
UPCR (g/gCr)	1.52 (0.35–6.50)	1.88 (0.54–4.72)	0.876
Urine protein (g/L)	1.70 (0.52–8.04)	1.59 (0.68–3.66)	0.507
Urine creatinine (g/L)	1.65 (0.92–2.34)	1.02 (0.79–1.40)	< 0.001
CKD stages			
stages 1–3	66 (94.29%)	64 (86.49%)	0.114
stages 4–5	4 (5.71%)	10 (13.51%)	
Ultrasonic renal length (cm)	10.61 ± 0.77	10.28 ± 0.90	0.018
Comorbidity			
Hypertension	9 (12.86%)	35 (47.30%)	< 0.001
Cardiovascular disease	1 (1.43%)	5 (6.76%)	0.210

Categorical variables are presented as n (%) and continuous variables as mean ± standard deviation or median (interquartile range) as appropriate

*BMI* body mass index, *eGFR* estimated glomerular filtration rate, *UPCR* urine protein to creatinine ratio

### Association between renal length and renal fibrosis across varied models

Table 2 displays the correlation between renal length and renal fibrosis through the application of multivariable logistic regression and GAM analysis. In the crude model, with each centimeter increase in renal length, the odds of having moderate-severe fibrosis decreased by 38% (odds ratio (OR): 0.62; 95% CI 0.41–0.93). After adjustment for demographic features, the association between the above-mentioned variables remained stable (OR: 0.61; 95% CI 0.39–0.97). Even after adjusting for all potential confounders, the relationship remained virtually unchanged (OR: 0.58; 95% CI 0.33–1.00).

**Table 2:** Relationship between renal length and moderate-severe renal fibrosis risk across models

Variable	Model 1		Model 2		Model 3	
	OR (95% CI)	*P* value	OR (95% CI)	*P* value	OR (95% CI)	*P* value
Renal length (cm)	0.62 (0.41–0.93)	0.020	0.61 (0.39–0.97)	0.037	0.58 (0.33–1.00)	0.048

Model 1 is unadjusted; Model 2 is adjusted for age, sex, and BMI; and Model 3 is adjusted for confounders in Model 2 and blood urea nitrogen (smooth adjustment), urine protein to creatinine ratio (smooth adjustment), hypertension, and cardiovascular disease

*OR* odds ratio, *CI* confidence interval

**Fig. 2:**
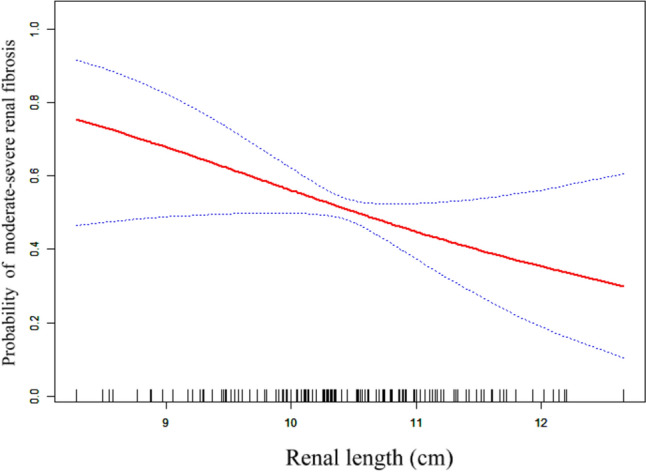
Association between renal length and the probability of moderate-severe renal fibrosis in non-diabetic CKD patients. An inverse linear association between renal length and moderate-severe renal fibrosis risk was found (*P* = 0.048) in a generalized additive model (GAM). The solid red line represents the smooth curve fit between these two variables. Blue bands represent the 95% confidence intervals from the fit. The GAM is adjusted for age, sex, BMI, blood urea nitrogen, urine protein to creatinine ratio, hypertension, and cardiovascular disease

To further investigate the relationship between renal length and renal fibrosis, we performed a fully adjusted smooth curve fitting analysis. As illustrated in Fig. 2, an inverse linear relationship exists between renal length and the risk of moderate-severe renal fibrosis. However, visual inspection alone is insufficient to fully capture potential nonlinear characteristics within the data. Consequently, we employed a nonlinear GAM to rigorously assess the possibility of nonlinear inflection points in this association. Using a recursive algorithm, an inflection point at a renal length of 9.37 cm was identified, indicating that the risk of fibrosis may vary at different ranges of renal length. Following this, we constructed piecewise linear models for the segments before and after the inflection point, and compared these models to a standard binary logistic regression model to assess the value of the piecewise approach. As shown in Table S2, the piecewise linear models did not achieve statistical significance (pre-inflection model, *P* = 0.164; post-inflection model, *P* = 0.207). Furthermore, a log-likelihood ratio test comparing the standard logistic regression model with the piecewise model indicated no significant difference (*P* = 0.057). These findings reinforce the linearity suggested by the fully adjusted smooth curve fitting analysis in Fig. 2, confirming the inverse linear relationship between renal length and the likelihood of moderate-severe renal fibrosis. In addition, the results support the adequacy of using a standard binary logistic regression model to describe this relationship.

Similar analyses were performed on both unadjusted and minimally adjusted models, yielding consistent results. The lack of statistical significance in the piecewise models further confirms the stability of the inverse linear relationship between renal length and fibrosis risk across varying adjustment levels. Detailed results can be found in Tables S3-S4 and Figures S1-S2.

### Diagnostic performance of renal length in different models

Renal length discriminates unsatisfactorily between mild fibrosis and moderate-severe fibrosis, as shown in Table S5. In the crude model, a renal length cut-off value of 10.14 cm yielded a sensitivity, specificity, and accuracy of 0.76, 0.49, and 0.62, respectively, with an AUC of 0.61 (95% CI 0.51–0.70). Even after adjusting for minimal and full covariates, the diagnostic performance of renal length remained suboptimal. In the minimally adjusted model, a cut-off value of 10.26 cm produced sensitivity, specificity, and accuracy values of 0.70, 0.50, and 0.60, respectively, with an AUC of 0.62 (95% CI 0.50–0.72). Similarly, in the fully adjusted model, a cut-off value of 10.32 cm resulted in sensitivity, specificity, and accuracy values of 0.61, 0.54, and 0.58, respectively, with an AUC of 0.58 (95% CI 0.45–0.70). The comparison of ROC curves is demonstrated in Figure S3.

## Discussion

In this prospective cross-sectional investigation, we explored the correlation between ultrasonic renal length and renal fibrosis in non-diabetic CKD patients. Our findings reveal an inverse relationship, indicating that greater renal length correlates with a reduced likelihood of moderate-severe renal fibrosis. However, it is crucial to note that renal length alone lacks adequate discriminatory power to serve as a clinical diagnostic indicator for determining renal fibrosis severity.

Renal fibrosis is histologically characterized by the presence of interstitial fibrosis, tubular cell apoptosis, and glomerular sclerosis. These pathologic changes result in the development of renal scars and progressive renal atrophy, which are typical morphologic manifestations [31]. Therefore, evaluating renal morphometric parameters and renal blood perfusion using renal US is a valuable approach to assessing both morphologic and hemodynamic alterations. Through this noninvasive method, the severity of renal fibrosis in patients with CKD can be indirectly determined [32].

Previous research has provided evidence that US findings of a thin cortex with increased echogenicity are indicative of irreversible damage, whereas a thick cortex with normal echogenicity may suggest reversible damage [33]. Nonetheless, due to the limited ability to differentiate renal cortex and medulla in patients with CKD, measuring cortical thickness using US is prone to significant variability among different operators as well as within the same operator [34]. In addition, accurate evaluation of renal parenchyma is hindered by the presence of kidney cysts, which are frequently observed in CKD patients and pose significant clinical challenges [35]. Another study reported that increased renal echogenicity can aid in the identification of individuals with advanced irreversible CKD in the context of glomerular disease [36]. This was achieved by comparing the renal parenchymal echogenicity with that of the liver parenchyma to determine the extent of kidney damage. However, advanced kidney disease frequently coexists with liver disease [37]. In cases where the liver parenchymal echogenicity is altered due to liver disorders, this evaluation method may become unreliable. The renal resistive index (RI), obtained from the renal interlobar artery, functions as a valuable indicator of kidney abnormalities and exhibits associations with CKD stages, albuminuria, and kidney parenchymal diseases such as intrarenal arteriosclerosis [38, 39]. However, recent studies suggest that a high renal RI is primarily influenced by systemic vascular parameters, specifically systemic arterial compliance, rather than kidney histology [40, 41]. US measurement of renal dimensions, specifically renal length, is a standard practice in clinical settings and serves as a commonly used surrogate marker for assessing renal pathologic damage. A prior study demonstrated that both intra- and inter-operator reproducibility of US-based renal length measurements in patients with CKD is considerably high and comparable to those obtained in magnetic resonance imaging [42]. Consequently, renal length measurement is a reliable methodology for evaluating the extent of renal pathologic damage, and it is routinely employed by radiologists as a US indicator to assess kidney damage.

A previous study reported a statistically significant correlation between renal length and the prevalence of glomerular sclerosis or tubular atrophy [43]. These findings imply an association between renal fibrosis and renal length. However, another study demonstrated that renal length was weakly correlated with kidney pathologic findings, and had limited value in guiding the decision of renal biopsy [44]. To be noted, aging, body mass, or concomitant illnesses could modify the morphology of kidney [45–47]. It would be difficult to identify the true association of renal length with renal fibrosis without adequately controlling for these potential confounding factors, which might be the reason for the conflicting results in previous studies. In the present study, we attempted to control for these potential underlying confounders and found that moderate-severe renal fibrosis risk decreases in CKD by 42% for each centimeter increase in renal length. This true inverse linear relationship between variables was confirmed through curve fitting via GAM models.

To further investigate the practical and clinical utility of renal length, we assessed its diagnostic capacity using covariate-adjusted ROC curve analysis [27]. The covariate-adjusted ROC curve analysis possesses the fundamental advantages inherent in traditional univariate ROC curve analysis [48]. First, it effectively enables the evaluation and comparison of diagnostic variables with differing fundamental units. Second, it comprehensively demonstrates the diagnostic variable’s classification capabilities across various threshold values. Furthermore, in the presence of an association between covariates and the diagnostic variable, covariate-adjusted ROC curve analysis proves to be effective in mitigating bias, an aspect not achievable through traditional univariate ROC curve analysis. In comparison to covariate-specific ROC curve analysis, covariate-adjusted ROC curve analysis offers distinct advantages. When covariates assume diverse values, the application of covariate-specific ROC curve analysis leads to the generation of multiple ROC curves, which complicates the interpretation of results [28]. Conversely, covariate-adjusted ROC curve analysis produces a unified ROC curve that better manages and interprets the analysis’ outcomes. In addition, in studies with a small sample size, covariate-specific ROC curve analysis frequently encounters issues of inadequate estimation accuracy, which can be effectively mitigated through covariate-adjusted ROC curve analysis. Upon thorough analysis, it was ultimately determined that renal length lacked sufficient diagnostic capability in assessing degrees of renal fibrosis after controlling the potential confounders, yielding an AUC of 0.58 (95% CI: 0.45–0.70) with limited sensitivity (0.61) and specificity (0.54), which meant that renal length could reflect the severity of renal fibrosis to a certain extent but was far from being a valuable diagnostic indicator. These findings underscore the necessity for physicians to exercise caution when utilizing renal length as an indicator for the management of CKD or as a determining factor for renal biopsy.

When comparing renal length to other established markers, such as eGFR, it is evident that eGFR remains a more accurate predictor of renal fibrosis, with a diagnostic AUC of 78.2% [49], clearly outperforming renal length as an independent marker. In addition, novel renal tubular markers, such as urinary retinol-binding protein (uRBP), have shown promise as indicators for assessing renal fibrosis [50]. However, both eGFR and these tubular markers require the collection of blood or urine samples and involve complex biochemical assays, making them somewhat invasive and cumbersome for routine clinical use [51]. In contrast, renal length, measured via routine US, is a non-invasive and easily accessible parameter in clinical practice, requiring no complex procedures. Nevertheless, given the limited diagnostic performance of renal length observed in this study, it is advisable not to rely solely on this parameter for fibrosis assessment. Instead, combining renal length with other US features and biochemical markers may provide a more comprehensive and accurate evaluation of renal fibrosis.

The study has several strengths. First, the outcome indicator in our study was based on pathologic evidence rather than eGFR. While eGFR or albuminuria are indicative of acute and chronic glomerular function changes, tubular atrophy and interstitial fibrosis are signs of chronic damage progression [52], which remain hidden in certain patients without clear alterations of functional markers. Consequently, relying on eGFR or CKD stage as a means to reflect the degree of kidney damage, followed by exploring the association between US parameters and the extent of kidney damage, as commonly performed in previous studies, is not a reliable approach [53]. Second, a rigorous statistical strategy was used to minimize the effects of residual confounders on the results of both risk association and diagnostic performance analyses. To investigate the nature of the relationship between variables, a thorough GAM analysis was conducted, encompassing both linear and nonlinear associations. Furthermore, a covariate-adjusted ROC curve analysis was employed to evaluate renal length diagnostic performance.

Our study also has some limitations. First, the number of cases with severe renal fibrosis was small, which limited our ability to perform stratified analysis between moderate and severe fibrosis groups. As a result, we merged these two groups for analysis to align with clinical practice, where both groups follow similar management strategies focused on reducing complications and delaying the need for dialysis or kidney transplantation. Future studies will aim to include a larger sample size, potentially through multi-center clinical research, to allow for more refined stratified analyses and better statistical power. Second, we did not investigate other US parameters, like renal cortical thickness or parenchymal echotexture, as the former had difficulty accurately and reproducibly measuring thickness and the latter lacked quantification metrics. In addition, the kidney’s maximum area and volume were not measured in this study, but these will be included in future analyses using three-dimensional US imaging and automated quantification techniques, which will allow for a more comprehensive evaluation of renal fibrosis.

## Conclusion

Our study elucidates a significant negative linear correlation between renal length and the likelihood of moderate-severe renal fibrosis in non-diabetic CKD patients, and thus renal length can be used as a simple indicator in the non-diabetic advanced CKD. However, the correlation is insufficient and this underscores the necessity for incorporating additional diagnostic parameters to enhance the accuracy and reliability of fibrosis assessment in CKD. Future research should focus on developing multifaceted diagnostic models that integrate renal length with other clinical, biochemical, and imaging markers to improve the precision of fibrosis evaluation and ultimately aid in better clinical management of CKD.

## Supplementary Information

Below is the link to the electronic supplementary material.

Supplementary file1 (DOCX 129 KB)

## Data Availability

The data presented in this study are available from the corresponding author upon reasonable request. Data is not publicly available due to privacy or ethical concerns.
