# Supplementary material for: Ultrasonic renal length as an indicator of renal fibrosis severity in non-diabetic patients with chronic kidney disease
Source: Clin Exp Nephrol. 2024 Nov 19;29(4):460–8. doi: 10.1007/s10157-024-02598-0 (PMC11937224; doi:10.1007/s10157-024-02598-0)
Supplement: Supplementary file 1 — Supplementary file1 (DOCX 129 KB) [file 10157_2024_2598_MOESM1_ESM.docx]

Table S1. Etiology of CKD

| Diagnosis | Number |
| --- | --- |
| IgA nephropathy  Membranous nephropathy  Minimal change nephropathy  Mesangial proliferative glomerulonephritis  Lupus nephritis  Focal segmental glomerular sclerosis  Others  Unknowns | 68 (47.2%)  32 (22.2%)  15 (10.4%)  9 (6.3%)  8 (5.6%)  8 (5.6%)  3 (2.1%)  1 (0.6%) |

Notes: Categorical variables are presented as n (%).

Abbreviations: CKD, chronic kidney disease.

Table S2. Results of standard linear regression model and two-piecewise linear regression model in fully adjusted modeling

| Outcome | Probability of Moderate-Severe renal fibrosis | |
| --- | --- | --- |
|  | OR (95% CI) | *P* value |
| Fitting model by standard logistic regression | 0.58 (0.33-1.00) | 0.048 |
| Fitting model by two-piecewise logistic regression |  |  |
| Inflection point of renal length (cm) | 9.37 | |
| ≤ inflection point | 0.10 (0.00-2.53) | 0.164 |
| > inflection point | 0.67 (0.36-1.25) | 0.207 |
| *P* for log-likelihood ratio test |  | 0.057 |

Notes: Adjusted for age, sex, BMI, blood urea nitrogen (smooth adjustment), urine protein to creatinine ratio (smooth adjustment), hypertension, and cardiovascular disease.

Abbreviations: OR, odds ratio; CI: confidence interval.

Table S3. Results of standard linear regression model and two-piecewise linear regression model in unadjusted modeling

| Outcome | Probability of Moderate-Severe renal fibrosis | |
| --- | --- | --- |
|  | OR (95% CI) | *P* value |
| Fitting model by standard logistic regression | 0.62 (0.41-0.93) | 0.020 |
| Fitting model by two-piecewise logistic regression |  |  |
| Inflection point of renal length (cm) | 10.53 | |
| ≤ inflection point | 0.38 (0.17-0.85) | 0.018 |
| > inflection point | 1.04 (0.47-2.34) | 0.915 |
| *P* for log-likelihood ratio test |  | 0.148 |

Abbreviations: OR, odds ratio; CI: confidence interval.

Table S4. Results of standard linear regression model and two-piecewise linear regression model in minimally adjusted modeling

| Outcome | Probability of Moderate-Severe renal fibrosis | |
| --- | --- | --- |
|  | OR (95% CI) | *P* value |
| Fitting model by standard logistic regression | 0.61 (0.39-0.97) | 0.037 |
| Fitting model by two-piecewise logistic regression |  |  |
| Inflection point of renal length (cm) | 9.30 | |
| ≤ inflection point | 0.07(0.00-2.28) | 0.133 |
| > inflection point | 0.73 (0.43-1.24) | 0.243 |
| *P* for log-likelihood ratio test |  | 0.159 |

Notes: Adjusted for age, sex, and BMI.

Abbreviations: OR, odds ratio; CI: confidence interval.

Table S5. Diagnostic performance of renal length in different models

| Index | Cut-off (cm) | AUC (95% CI) | Sensitivity | Specificity | Accuracy |
| --- | --- | --- | --- | --- | --- |
| Model 1 | 10.14 | 0.61 (0.51-0.70) | 0.76 | 0.49 | 0.62 |
| Model 2 | 10.26 | 0.62 (0.50-0.72) | 0.70 | 0.50 | 0.60 |
| Model 3 | 10.32 | 0.58 (0.45-0.70) | 0.61 | 0.54 | 0.58 |

Notes: Model 1 is unadjusted; Model 2 is adjusted for age, sex, and BMI; and Model 3 is adjusted for confounders in Model 2 and blood urea nitrogen, urine protein to creatinine ratio, hypertension, and cardiovascular disease.

Abbreviations: AUC, area under the curve; CI: confidence interval.


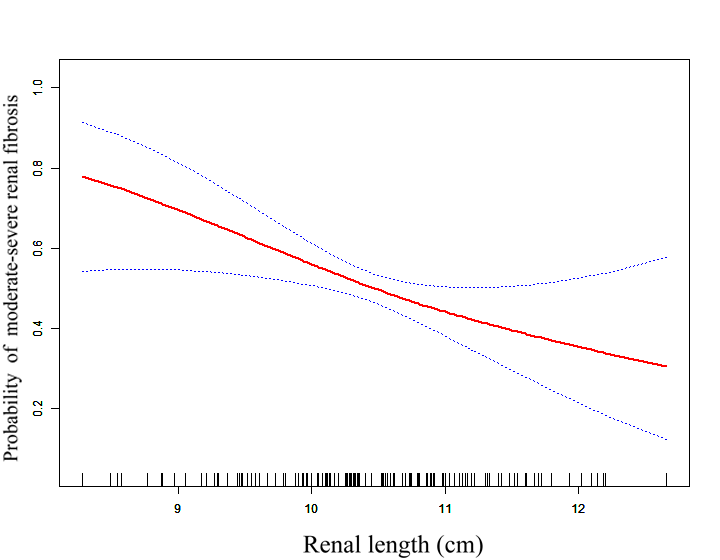

Figure S1. Association between renal length and renal fibrosis in unadjusted modeling. An inverse linear association between renal length and the risk of moderate-severe renal fibrosis was found (*P* = 0.020) in a generalized additive model. The solid red line represents the smooth curve fit between variables. Blue bands represent the 95% confidence intervals from the fit.


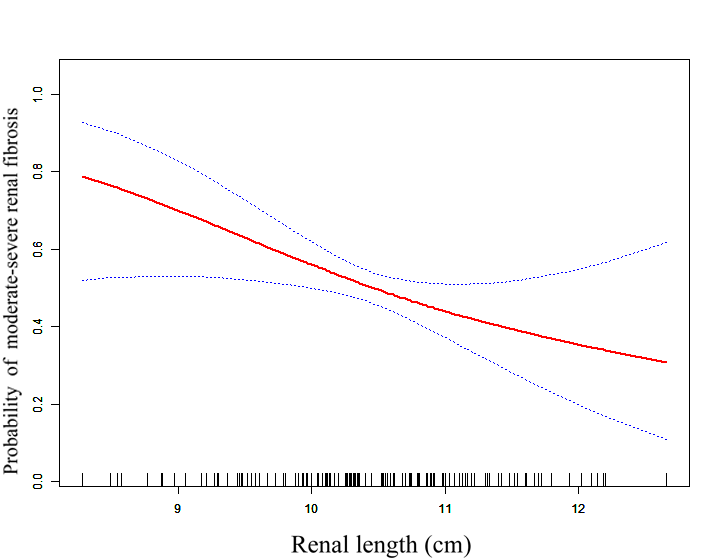

Figure S2. Association between renal length and renal fibrosis in minimally adjusted modeling. An inverse linear association between renal length and the risk of moderate-severe renal fibrosis was found (*P* = 0.037) in a generalized additive model. The solid red line represents the smooth curve fit between variables. Blue bands represent the 95% confidence intervals from the fit. All values are adjusted for age, sex, and BMI.


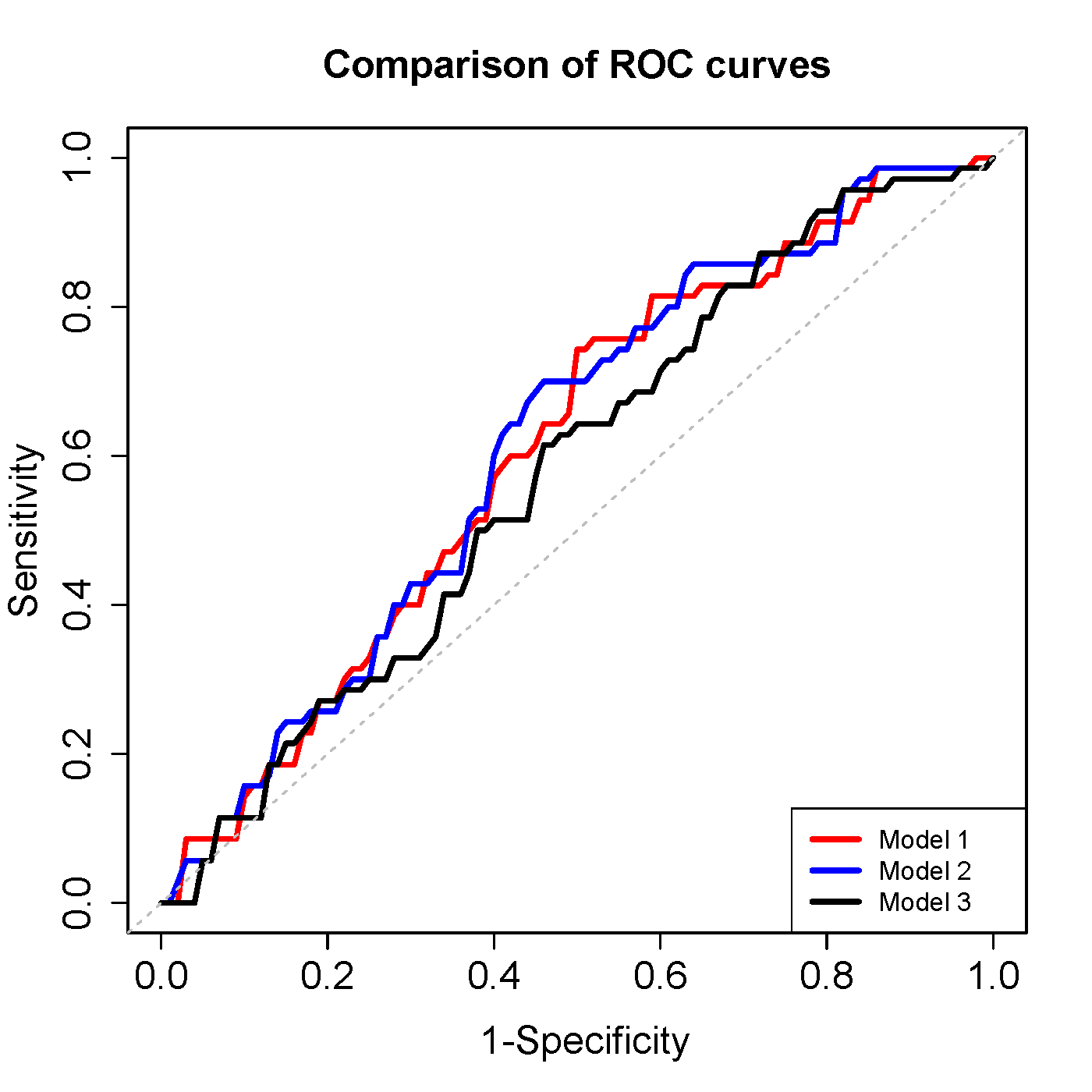


Figure S3. Receiver operating characteristic curves based on renal length in different adjusted models when differentiating moderate-severe renal fibrosis from mild. Model 1 is unadjusted; Model 2 is adjusted for age, sex, and BMI; and Model 3 is adjusted for confounders in Model 2 and blood urea nitrogen, urine protein to creatinine ratio, hypertension, and cardiovascular disease.
